# Relationship between Mild Cognitive Impairment, Pre-Frailty, Physical and Psychological Functioning, and Functional Capacity among Community-Dwelling Older Adults

**DOI:** 10.3390/healthcare11182542

**Published:** 2023-09-14

**Authors:** Tsuyoshi Katsurasako, Shin Murata, Akio Goda, Kayoko Shiraiwa, Jun Horie, Teppei Abiko, Hideki Nakano

**Affiliations:** 1 Graduate School of Health Sciences, Kyoto Tachibana University, Kyoto 607-8175, Japan; 2 Department of Rehabilitation, Koka City Minakuchi Medical Care Center, Koka 528-0049, Japan; 3 Department of Physical Therapy, Faculty of Health Sciences, Kyoto Tachibana University, Kyoto 607-8175, Japan; murata-s@tachibana-u.ac.jp (S.M.); shiraiwa@tachibana-u.ac.jp (K.S.); horie-j@tachibana-u.ac.jp (J.H.); abiko@tachibana-u.ac.jp (T.A.); nakano-h@tachibana-u.ac.jp (H.N.); 4 Department of Physical Therapy, Faculty of Health and Medical Sciences, Hokuriku University, Kanazawa 920-1180, Japan; goda@hokuriku-u.ac.jp

**Keywords:** community-dwelling older adults, mild cognitive impairment, pre-frailty

## Abstract

Mild cognitive impairment (MCI) is associated with a high risk of dementia. Reportedly, 54.6% of older adults with MCI fall into the pre-frailty category. However, it is unclear what differences exist in older adults with regard to their physical, psychological, and functional capacities, which depend on MCI, pre-frailty, or a combination of the two. This study aimed to examine the differences between the association between physical function, psychological function, and functional capacity by examining a combination of MCI and pre-frailty among community-dwelling older Japanese individuals. The participants in the analysis were 236 older people living in the community. They were classified into four groups, as follows: normal, MCI only, pre-frailty only, and pre-frailty/MCI; furthermore, their physical, psychological, and functional capacities were compared. In addition, a multinomial logistic regression analysis was performed using MCI and pre-frailty as dependent variables. No associated factors were identified for MCI only, and a depressive mood was only associated with pre-frailty. When pre-frailty and MCI were combined, knee extension muscle strength, fastest gait speed, the s30 s chair stand test, depressed moods, and the instrumental activities of daily living (IADL) were correlated. Our results suggest that when MCI and pre-frailty are combined, declines in both physical and psychological functions and IADL are likely to occur.

## 1. Introduction

Dementia significantly impacts society, increasing the burden on families and caregivers and causing a loss in terms of economic productivity. The incidence of dementia is rapidly increasing worldwide, and the number of patients with dementia is projected to reach 82 million by 2030 [1]. Japan is one of the world’s most hyper-aged societies, with 28.9% of its total population aged ≥65 years as of 2021 [2]. As it is estimated that dementia will continue to increase in accordance with the aging population, the early prevention of dementia is important to extend healthy life expectancies.

Mild cognitive impairment (MCI), which is considered to be a precursor state to dementia, has been the focus of attention with regard to the early prevention of dementia. MCI is a condition in which a person has no diagnosis of dementia and can independently conduct daily activities, but it is not considered age-appropriate [3]. Older individuals with MCI are at a high risk of progressing to dementia [4,5], but they are also expected to recover from MCI. Iraniparast et al. [6] reported that 30.3% of older individuals with MCI recovered. Notably, when patients have MCI, this is an important period for dementia prevention, and it is necessary to examine the factors associated with MCI to obtain specific guidelines for the early prevention of dementia.

Previous studies [7,8,9] examining the factors associated with MCI have reported that MCI is associated with decreased physical functions such as grip strength, the timed up and go test (TUG) as an index of composite walking ability, and gait speed. It has also been reported that low grip strength and slow gait speed affect MCI after 10 years [10].

Conversely, it has been reported that MCI is not associated with physical functions such as grip strength, TUG, and gait speed [11,12]. It has also been reported that some MCI patients are mildly impaired when conducting instrumental activities of daily living (IADL), which are higher-order activities pertaining to daily living skills; although, some MCI patients maintain their ability to carry out IADL [13]. Thus, there are reports which state that MCI is associated with physical function and IADL, and vice versa. Hence, there is no consensus of opinion.

Nyunt et al. [14] reported that 54.6% of older adults with MCI were in a state of pre-frailty, which is the stage before frailty. Therefore, previous studies examining relationships between physical and psychological functions and functional capacity in older adults with MCI may have included patients with concomitant pre-frailty. Importantly, pre-frailty increases the risk of transitioning to frailty [15], and frailty and pre-frailty increase the risk of cognitive impairment [16]. The combined state of cognitive impairment and pre-frailty increases the risk of dementia and mortality [17]. Therefore, for the early prevention of dementia, interventions targeting MCI, the precursor state of dementia, and pre-frailty, the stage before frailty, are considered effective. It is therefore important to clarify how MCI only, pre-frailty only, and MCI with pre-frailty relate to physical and psychological functions and functional capacity, respectively. However, although MCI and pre-frailty have been reported to be associated with a risk of dementia and death [17], few reports have compared physical function with MCI in pre-frailty and healthy individuals. Moreover, it is unclear whether physical functions are associated with grip strength, walking ability, and balance. Furthermore, patients exhibiting MCI and frailty have been reported to be at a higher risk of struggling with IADL [18,19], but the association between MCI, pre-frailty, and IADL is unclear, with few reports [20].

We hypothesized that patients who have MCI with frailty, and MCI with pre-frailty, would be more likely to exhibit decreased physical function, and they would be less able to carry out IADL than patients who have MCI or pre-frailty alone. This study aimed to examine the relationship between physical and psychological functions and functional capacity, with regard to MCI and pre-frailty in independent, community-dwelling Japanese older adults. 

## 2. Materials and Methods

### 2.1. Subjects

**Figure 1:**
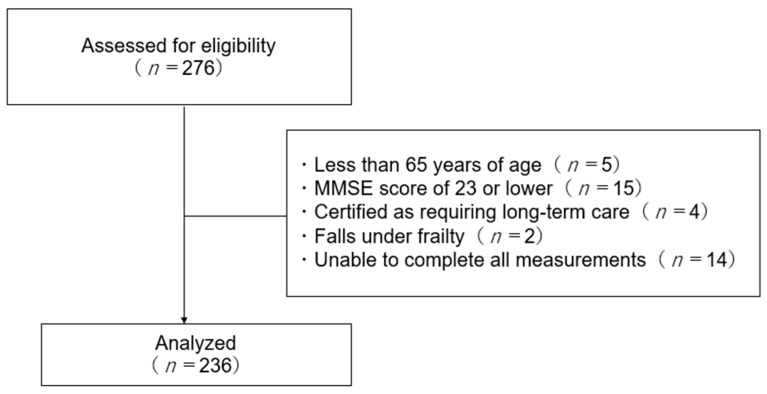
Flowchart of participation criteria.

The participants of this study were 276 older adults who took part in physical fitness tests that were held in three regions of Japan. Physical fitness measurement sessions were held in three regions in September 2022. The participants lived in an area sponsored by the local government, and they voluntarily participated in the physical fitness test. The duration of each physical fitness measurement session was one week. The venue for the physical fitness test was a civic center with a wide, level-free space that facilitated the evaluation of physical functions. The inclusion criteria for the participants were as follows: community-dwelling older adults who were able to voluntarily participate in a local government-sponsored physical fitness test, age ≥65 years, a Mini-Mental State Examination (MMSE) score of 24 or higher, and a measure of cognitive function. Exclusion criteria were those certified as requiring long-term care, frail, or unable to complete all measurements. Of the participants, 236 met the inclusion criteria and were included in the analysis (Figure 1).

This study was approved by the Ethics Committee of Kyoto Tachibana University (Approval No. 18–26). The purpose and objectives of the study were explained to the participants in advance, and those who provided consent were included in the study. Ethical considerations based on the Declaration of Helsinki were fully taken into account.

### 2.2. Determination of MCI

Cognitive function was assessed using the internationally widely used MMSE [21]. The MMSE is widely used as a tool to screen for global cognitive impairment. The MMSE consists of 11 items related to orientation (date and time, place), memorization, calculation, recall, naming, repetition, three-step command, reading comprehension, writing, and structure. The score is 0 to 30, and a higher score indicates a higher level of cognitive function. According to previous studies, cognitive decline or dementia is suspected if the total MMSE score is 23 points or fewer [22]. Moreover, a score of 27 or less may indicate mild cognitive decline [23]. Therefore, in this study, MMSE scores of 28 points or more were judged normal, patients with 27 to 24 points were considered to have MCI, and those who scored below 23 points were excluded.

### 2.3. Determination of Pre-Frailty

Pre-frailty was determined using the revised Japanese version of the Cardiovascular Health Study (CHS) [24] criteria. Five items were evaluated, as follows: (1) decreased grip strength (less than 28 kg in men and less than 18 kg in women); (2) normal gait speed (less than 1.0 m/s); (3) weight loss; (4) fatigue (feeling tired without any reason in the past two weeks); and (5) decreased activity (no light exercise or regular exercise or sports). If none of the five items of the CHS were true, the participants were judged to be healthy, and if one or two were true, the participants were judged to be in a pre-frailty state. Therefore, in this study, 0 cases were judged to be normal, 1 to 2 cases were judged to be pre-frailty, and 3 or more cases were excluded.

### 2.4. Group Classification

The participants were classified into four groups, depending on the presence or absence of MCI and a pre-frailty condition, as follows: normal (CHS = 0, MMSE ≥ 28), pre-frailty only (1 ≤ CHS ≤ 2, MMSE ≥ 28), MCI only (CHS = 0, 24 ≤ MMSE ≤ 27), and pre-frail/MCI (1 ≤ CHS ≤ 2, 24 ≤ MMSE ≤ 27).

### 2.5. Physical Functions

Grip strength [25] was measured using a digital grip strength meter (T.K.K.58401; Takei Scientific Instruments Co., Ltd., Niigata, Japan), adjusted such that the second joint of the index finger was at a right angle. The limbs were placed in a natural position with the arms hanging down, and measurements were taken twice on each side. The maximum value was considered to be the measured value.

Knee extension muscle strength [26] was measured using a handheld dynamometer (μ-Tas F-1; Anima Corp., Tokyo, Japan) with a belt connected to a chair post in a seated-chair position, and isometric muscle strength was measured in the lower leg drop position. Measurements were taken with the hip and knee joints in a 90 degree flexion, twice on each side. The maximum value was considered to be the measured value.

Normal walking speed was measured by setting up a 5 m walking path (measurement section) and a 3 m auxiliary path at both ends. The participants were instructed to walk at a normal speed, and the time taken was measured using a digital stopwatch.

The fastest walking speed [27] was measured by setting up a 5 m walking path (measurement section) and a 3 m supporting path at both ends, and the time taken was measured using a digital stopwatch. The participants were instructed to walk as fast as possible with maximum effort.

The TUG test [28] was performed by measuring the time taken to stand up from a seated-chair position that was approximately 40 cm in height; then, the participants were required to move forward as fast as possible with maximum effort, turn around a landmark placed 3 m ahead, and sit down in the chair again.

During the 30 s chair–stand test (CS-30) [29], a chair that was approximately 40 cm high was used, and the starting point for the test required the participant to sit with arms crossed in front of the chest. CS-30 is used not only for leg muscle strength, but also to evaluate composite motor function when a participant stands up from a chair [30]. The number of times the participant was able to stand for 30 s was used as the measurement value.

During the open-eye one-leg standing time measurement [31], a digital stopwatch was used to measure the time from when the participant raised one leg to when the foot touched the ground with both hands on the side of the body while barefoot. The maximum time taken was 60 s, which was measured twice on each side. The longest measurement time was used as the final measurement time.

### 2.6. Psychological Function

Psychological function was assessed using the Geriatric Depression Scale-5 (GDS-5) [32], a shortened version of the Geriatric Depression Scale for the Elderly. The GDS-5 is a scale for evaluating depressed moods, and the five evaluation items are life satisfaction, boredom, going out, motivation to live, and helplessness. A self-administered questionnaire was used to answer “yes” or “no” to each item, and the total score was calculated (0 to 5).

### 2.7. Functional Capacity

Functional capacity was assessed using five questions [33] from the “Activities of Daily Living” domain on the basic checklist. The basic checklist, developed by the Ministry of Health, Labor, and Welfare in Japan, is a simple self-reporting yes/no survey consisting of 25 questions. It is extensively used to assess seniors’ physical and mental functions and functional capacity, and it is also used to identify older adults who are at risk of requiring support or care in the near future [33]. For 5 questions in the ‘Activities of Daily Living’ domain, 1 point was added if there was a problem with the IADL. In this study, IADL decline was defined as a score of 1 or more in the “Activities of Daily Living” domain.

### 2.8. Other Items

Other evaluated items included age, sex, height, weight, body mass index (BMI), educational history, and history of falls. Muscle mass was evaluated based on body composition. Muscle mass was measured using a portable body component analyzer, InBody 470 (InBody Japan Inc., Tokyo, Japan) [34], based on the bioelectrical impedance method. The skeletal muscle mass index (SMI) was calculated by dividing the skeletal muscle mass of the extremities obtained from the measurement using the square of height.

### 2.9. Statistical Analysis

In this study, the participants were classified into four groups, depending on the presence or absence of MCI and pre-frailty, as follows: normal, pre-frailty only, MCI only, and pre-frail/MCI. To compare the physical, psychological functioning, and functional capacity of the four groups, the participants’ age, sex, educational background, height, weight, BMI, SMI, knee extension muscle strength, fastest gait speed, TUG, CS-30, open-eyed one-leg standing time, GDS-5 score, presence or absence of declining ability to conduct IADL, and history of falls were considered. Grip strength and normal gait speed were not included in the comparison of the four groups because they were included in the CHS criteria.

First, the normality of the data was checked using the Shapiro–Wilk test for age, educational history, height, weight, BMI, SMI, knee extension muscle strength, fastest gait speed, TUG, CS-30, open-eyed one-leg standing time, and GDS-5. One-way analysis of variance (ANOVA) was performed on normally distributed data. For items with statistically significant differences, multiple comparisons were performed using the Bonferroni correction test. For non-normally distributed data, the Kruskal–Wallis test was performed. For items with statistically significant differences, multiple comparisons were performed using the Dunn test. χ^2^ tests were performed for the categorical variables of sex, history of falls, and presence of ability to conduct IADL, and multiple comparisons were performed using the Bonferroni correction test for items that were significantly different.

Next, a multinomial logistic regression analysis was performed to identify what factors were associated with pre-frailty only, MCI only, and pre-frail/MCI groups, as compared with the normal group. The dependent variables were the normal group, which was the reference group, the pre-frailty-only group, the MCI-only group, and the pre-frail/MCI group. The independent variables were items (age, knee extension muscle strength, fastest walking speed, TUG, CS-30, GDS-5, and IADL) that showed significant differences between the four groups. The null hypothesis was “This independent variable should not be included in the regression equation”, and if the significance probability was less than 0.050, the null hypothesis was rejected and “This independent variable may be included in the regression equation”. SPSS ver. 28.0 (IBM Japan, Ltd., Tokyo, Japan) and R ver. 4.1.2 (free software) were used for analysis, and the significance level was set at 5%.

## 3. Results

The four groups in this study comprised 89 (37.7%) participants in the normal group, 65 (27.5%) in the pre-frailty-only group, 40 (16.9%) in the MCI-only group, and 42 (17.8%) in the pre-frailty/MCI group. The mean MMSE scores were 29.4 ± 0.8 for the healthy group, 29.2 ± 0.9 for the pre-frailty-only group, 25.7 ± 1.0 for the MCI-only group, and 25.6 ± 1.0 for the pre-frailty/MCI group.

The most common pre-frailty items in the CHS criteria were fatigue (49.2%), gait speed (10.8%), and decreased activity (10.8%) in the pre-frailty-only group. The pre-frailty/MCI group showed the highest rates of fatigue (46.2%), gait speed (21.4%), and decreased activity (16.7%). The results of the comparison of each variable among the four groups are presented in Table 1. The four-group comparison showed significant differences in age, knee extension muscle strength, fastest gait speed, TUG test, CS-30, GDS-5, and IADL scores (*p* < 0.05). As a result of multiple comparisons, age was significantly higher in the pre-frailty/MCI group than in the normal group, and knee extension muscle strength was significantly lower in the pre-frailty/MCI group than in the normal group. TUG was significantly slower in the pre-frailty/MCI group than in the normal and MCI only groups. CS₋30 was significantly lower in the pre-frailty/MCI group than in the other groups. GDS-5 scores were significantly higher in the pre-frailty-only group and the pre-frailty/MCI group than in the normal group. The ability to conduct IADL was significantly higher in the pre-frailty/MCI group compared with the normal group.

Next, multinomial logistic regression analysis was performed, and no relevant factors were extracted for the MCI-only group. In the pre-frailty-only group, only the GDS-5 (odds ratio 1.952, 95% confidence interval 1.324–2.878) was extracted as an associated factor.

Conversely, in the pre-frailty/MCI group, knee extension muscle strength (odds ratio 0.923, 95% confidence interval 0.862–0.990), fastest gait speed (odds ratio 0.091, 95% confidence interval 0.009–0.878), CS-30 (odds ratio 0.873, 95% confidence interval 0.792–0.962), GDS-5 (odds ratio 1.642, 95% confidence interval 1.037–2.599), and IADL (odds ratio 3.544, 95% confidence interval 1.253–10.026) were identified as associated factors (Table 2). Age and TUG, which showed significant differences in multiple comparisons, were not extracted as related factors in the multinomial logistic regression analysis.

## 4. Discussion

This study aimed to clarify the relationship between physical function, psychological function, and functional capacity in four groups, depending on the presence or absence of MCI and pre-frailty. Multinomial logistic regression analysis showed that no relevant factors were extracted for the MCI-only group, and GDS-5 was extracted as a relevant factor for the pre-frailty-only group. Knee extension muscle strength, fastest gait speed, CS-30, GDS-5, and IADL were identified as relevant factors in the pre-frailty/MCI group. These findings suggest that MCI alone and pre-frailty are not associated with physical function and IADL, but when pre-frailty and MCI are combined, a decline in physical and psychological function, as well as the ability to conduct IADL, are associated.

Only the GDS-5 score was identified as an associated factor in the pre-frailty only group. A study concerning community-dwelling older participants reported that depressed moods affect frailty, in accordance with the CHS criteria [35]; these results are similar to those of the present study. In contrast, physical function and functional capacity were not associated with the pre-frailty only group. Chen et al. [17] noted that pre-frailty is a condition that meets only one or two of the frailty criteria, and it is not significantly associated with functional capacity impairment or other disabilities. Among the CHS criteria in this study, fatigue was the most common (49.2%) in the pre-frailty-only group, and approximately 10% of the participants fell into the categories of reduced gait speed and activity. Therefore, we inferred that no decline in physical function could interfere with activities of daily living.

No associated factors were identified in the MCI-only group. Previous studies have reported that MCI is associated with decreased physical functions, such as grip strength and TUG [7,8,9], and decreased gait speed is a predictor [10] of MCI. Conversely, there are reports that MCI is not associated with grip strength, walking speed, or TUG [11,12]. Nyunt et al. [14] reported that 54.6% of older adults with MCI had pre-frailty, but previous studies [7,8,9,10,11,12] did not consider the presence of frailty or pre-frailty in older adults with MCI. Therefore, we deduced that the MCI only group did not extract the relevant factors, and that MCI with pre-frailty was associated with reduced physical function.

In contrast, in the pre-frailty/MCI group, physical functions such as knee extension muscle strength, fastest gait speed, CS-30, psychological function of GDS-5, and decline in the functional capacity of IADL were extracted as relevant factors. Previous studies have shown that both frailty and cognitive impairment have cumulative adverse effects that substantially increase mortality risk and dementia [36,37]. In a previous study concerning older individuals with cognitive impairment and pre-frailty, and who had MMSE scores of 24 or less, physical functions such as the 6 min walking distance, TUG, and the ability to stand and sit five times reportedly decreased [38]. In this study, patients with MCI who had MMSE scores ranging from 27 to 24 points, were included, and it was inferred that the degree of cognitive decline was milder than that in previous studies [38]. Another study reported that half of the patients with MCI showed symptoms such as fatigue, low lean muscle mass, and low physical activity [14]. In contrast, pre-frailty has been associated with decreased cognitive functions such as memory and processing speed [39]. Walking involves complex cognitive functions, including executive function, spatial awareness, and memory [40]. Therefore, we inferred that the overlap between MCI and pre-frailty, even with mild cognitive decline, had a cumulative effect on physical function, resulting in reduced muscle strength and walking speed.

Regarding functional capacity, previous studies have reported that MCI with frailty increases the risk that one will be unable to conduct IADL, but regarding MCI with pre-frailty, this risk is unclear [20]. It has also been reported that some elderly patients with MCI have limited IADL impairment, whereas others maintain their ability to conduct IADL; thus, there is no consensus [13]. Our results showed that MCI and pre-frailty alone were not associated with an inability to conduct IADL, and only pre-frailty and MCI combined were significantly associated with an inability to conduct IADL. These findings suggest that MCI with frailty and MCI with pre-frailty may be associated with an inability to conduct IADL. Conversely, regarding MCI, a previous study reported that the rate at which the ability to conduct IADL declined in older adults with MCI was 24% [41]. From these results, it was inferred that the ability to conduct IADL is maintained by MCI alone, without pre-frailty, but physical function declines when pre-frailty and MCI overlap, and there is a high possibility that the ability to conduct IADL will be reduced.

The results of this study showed that no associated factors were extracted for MCI only and GDS-5 was associated with pre-frailty only. It has been suggested that when pre-frailty and MCI are combined, declines in both physical and psychological functions and ability to conduct IADL are observed. Furthermore, it has been reported [36,37] that the overlap between cognitive impairment and frailty increases the risk of progression to dementia. Therefore, it is important to intervene based on the characteristics of MCI-only, pre-frailty-only, and a combination of MCI and pre-frailty.

Regarding early interventions to prevent dementia, older adults with MCI need to strive to maintain their physical function and intervene to prevent pre-frailty. For older patients with cognitive function in the normal range, and who are in a pre-frail state, it is important to support them in improving their depressive tendencies and preventing them from developing MCI; this is because depression and cognitive dysfunction are strongly associated [42,43]. For older individuals with both MCI and pre-frailty status, it is important to maintain physical function to control the progression to frailty and improve depressive tendencies. In addition, since the pre-frailty/MCI group is also associated with a decline in the ability to conduct IADL, it is necessary to conduct IADL assessments even when patients are independent in their daily lives, and it is necessary to make efforts to detect inabilities to conduct IADL at an early stage.

This study had some limitations. First, this study used a cross-sectional design, and it was difficult to estimate causality between the presence or absence of MCI and pre-frailty and IADL. Further longitudinal research is required to investigate causality. Second, in this study, participants were divided into four groups, depending on the presence or absence of MCI and pre-frailty, and the sample size of each group was not sufficient. Therefore, it is conceivable that a β error may have occurred. Larger sample sizes are needed to clarify the factors associated with normal versus pre-frailty only, MCI only, and pre-frailty/MCI groups. Third, the participants recruited in this study were able to voluntarily participate in physical fitness tests and they were taken from a relatively healthy elderly population. It is unclear whether the results of this study apply to populations with poor health, and thus, similar studies are needed. Finally, the MMSE was used to determine MCI in this study, which may have resulted in false negatives. In the future, it will be necessary to consider using other cognitive function tests in combination with each other.

Despite these limitations, the results of this study are significant because they provide insight into the complex interplay between MCI, pre-frailty, and functional capacity.

## 5. Conclusions

In this study, we grouped participants into the normal, pre-frailty only, MCI only, and pre-frailty/MCI groups, and we compared the physical and psychological functions and functional capacities of older adults. The MCI only and pre-frailty only groups were not associated with reduced physical function or functional capacity. However, physical and psychological functions and the ability to conduct IADL were affected when MCI and pre-frailty were combined. To prevent dementia, older adults with MCI only should strive to maintain physical function. Regarding pre-frailty only patients, it is important to help reverse depressive tendencies to prevent the development of MCI. Older adults with both MCI and pre-frailty need to maintain their physical function to slow down the progression to frailty and improve their depressive tendencies. Furthermore, it is necessary to evaluate the ability to conduct IADL, even if the patient is independent in daily life. Thus, we should strive for the early detection of the inability to conduct IADL.

## Figures and Tables

**Table 1:** Comparison results of each variable among the four groups.

	A: Pre-Frailty/MCI (*n* = 42)	B: MCI only (*n* = 40)	C: Pre-Frailty only (*n* = 65)	D: Normal (*n* = 89)	*p*	Multiple Comparison
Age (years)	78.1 ± 4.9	77.3 ± 5.7	76.2 ± 6.7	74.9 ± 5.6	0.014	A > D ^#^
Gender (Male/Female)	9 × 33	10 × 30	14 × 51	18 × 71	0.946	
Education (years)	12 (9–12.5)	12 (9.3–14)	12 (12–14)	12 (12–14)	0.063	
Height (cm)	151.2 (147.8–158.8)	155.0 (147.0–157.3)	152.9 (146.5–160.4)	152.7 (148.9–157.2)	0.874	
Weight (kg)	50.8 ± 9.4	53.2 ± 9.0	51.6 ± 10.2	53.0 ± 10.2	0.552	
BMI (kg/m^2^)	22.0 ± 2.9	22.6 ± 3.0	21.7 ± 3.3	22.2 ± 3.4	0.628	
SMI (kg/m^2^)	5.96 (5.22–6.34)	6.22 (5.79–6.79)	6.04 (5.35–6.68)	5.93 (5.46–6.83)	0.279	
Knee extension strength (kgf)	21.1 (16.8–27.6)	23.8 (19.8–32.1)	23.8 (18.3–29.3)	26.0 (20.9–31.6)	0.007	A < D ^†^
Fastest gait speed (m/s)	1.66 (1.43–1.89)	1.86 (1.68–2.08)	1.80 (1.54–2.00)	1.87 (1.67–2.03)	0.003	A < B, D ^†^
TUG (s)	6.5 (6.0–7.0)	5.8 (5.5–6.7)	6.2 (5.4–7.1)	5.9 (5.3–6.3)	0.004	A > D ^†^
CS-30 (times)	17.6 ± 6.0	21.9 ± 5.9	21.3 ± 6.1	22.0 ± 5.3	<0.001	A < B, C, D ^#^
One-leg standing (s)	13.5 (7.6–35.8)	24.6 (8.5–60)	25.0 (9–60)	37.0 (15–60)	0.088	
GDS-5	0 (0–1.3)	0 (0–1)	1 (0–2)	0 (0–1)	<0.001	A, C > D ^†^
History of falls (yes/no)	12 × 30	7 × 33	13 × 52	14 × 75	0.373	
IADL decline (yes/no)	14 × 28	10 × 30	14 × 51	11 × 78	0.040	A > D ^#^

Age, weight, BMI, and CS-30 are mean ± standard deviation. Gender, history of falls, and IADL decline are frequencies. Education, length, SMI, knee extension strength, fastest gait speed, TUG, and open-eye standing. GDS-5 is the central value (quartile range). One-way ANOVA for age, weight, BMI, and CS-30. Gender, history of falls, and IADL are χ^2^ tests. The Kruskal–Wallis test was used for other items. ^#^ Bonferroni method. ^†^ Dunn test. BMI, Body Mass Index. SMI, Skeletal Muscle Mass Index. TUG, Timed up and go test. CS-30, 30 s chair–stand test. GDS-5, Geriatric Depression Scale-5. IADL, Instrumental Activities of Daily Living.

**Table 2:** Results of multinomial logistic regression analysis with reference to the normal group.

	Independent Variable	Odds Ratio	95% Confidence Interval	*p*
Pre-frailty only	Age	0.999	0.937–1.066	0.982
	Knee extension strength	0.987	0.944–1.031	0.554
	Fastest gait speed	0.606	0.114–3.208	0.556
	TUG	1.460	0.929–2.293	0.101
	CS-30	1.033	0.964–1.107	0.358
	GDS-5	1.952	1.324–2.878	<0.001
	IADL	1.540	0.607–3.907	0.364
MCI only	Age	1.063	0.990–1.141	0.092
	Knee extension strength	0.991	0.943–1.041	0.707
	Fastest gait speed	3.220	0.528–19.641	0.205
	TUG	1.476	0.886–2.457	0.135
	CS-30	1.017	0.942–1.097	0.668
	GDS-5	1.210	0.751–1.949	0.434
	IADL	2.128	0.781–5.799	0.140
Pre-frailty/MCI	Age	1.050	0.973–1.133	0.210
	Knee extension strength	0.923	0.862–0.990	0.025
	Fastest gait speed	0.091	0.090–0.878	0.038
	TUG	0.649	0.369–1.140	0.132
	CS-30	0.873	0.792–0.962	0.006
	GDS-5	1.642	1.037–2.599	0.034
	IADL	3.544	1.253–10.026	0.017

Model χ^2^ test *p* < 0.001. The independent variables are age, knee extension strength, Fastest gait speed, TUG, CS-30, GDS-5, and IADL. TUG, Timed up and go test. CS-30, 30 s chair–stand test. GDS-5, Geriatric Depression Scale-5. IADL, Instrumental Activities of Daily Living.

## Data Availability

Not applicable.
